# Pan-Tetris: an interactive visualisation for Pan-genomes

**DOI:** 10.1186/1471-2105-16-S11-S3

**Published:** 2015-08-13

**Authors:** André Hennig, Jörg Bernhardt, Kay Nieselt

**Affiliations:** 1 University of Tübingen, Center for Bioinformatics Tübingen, 72076 Tübingen, Germany; 2 University of Greifswald, Institute for Microbiology, 17487 Greifswald, Germany

**Keywords:** Gene Sequence analysis, Genomics, Bioinformatics Visualization, Software Visualization, Data Aggregation

## Abstract

**Background:**

Large-scale genome projects have paved the way to microbial pan-genome analyses. Pan-genomes describe the union of all genes shared by all members of the species or taxon under investigation. They offer a framework to assess the genomic diversity of a given collection of individual genomes and moreover they help to consolidate gene predictions and annotations. The computation of pan-genomes is often a challenge, and many techniques that use a global alignment-independent approach run the risk of not separating paralogs from orthologs. Also alignment-based approaches which take the gene neighbourhood into account often need additional manual curation of the results. This is quite time consuming and so far there is no visualisation tool available that offers an interactive GUI for the pan-genome to support curating pan-genomic computations or annotations of orthologous genes.

**Results:**

We introduce Pan-Tetris, a Java based interactive software tool that provides a clearly structured and suitable way for the visual inspection of gene occurrences in a pan-genome table. The main features of Pan-Tetris are a standard coordinate based presentation of multiple genomes complemented by easy to use tools compensating for algorithmic weaknesses in the pan-genome generation workflow. We demonstrate an application of Pan-Tetris to the pan-genome of *Staphylococcus aureus*.

**Conclusions:**

Pan-Tetris is currently the only interactive pan-genome visualisation tool. Pan-Tetris is available from http://bit.ly/1vVxYZT

## Background

Next-generation sequencing technologies have accelerated the pace at which whole genomes can be sequenced, opening the possibility to sequence a large number of individuals from one common species (such as the 1000 Genomes Project in Human [1], or the 1001 Genomes Project in *Arabidopsis thaliana *[2]). The genomes of individuals of one species are compared on different levels, ranging from single nucleotide variation up to chromosomal rearrangements. In addition, in bacteria individual strains within one species can show extensive variation in their gene content, such that either individual genes or larger clusters of genes can be lost or newly acquired by horizontal gene transfer. In particular, in pathogenic strains of a bacterial species the degree of virulence can be attributed to the absence or presence of genes. This latter observation has led to the coining of the term pan-genome, which traditionally encompasses the full repertoire of all genes of a bacterial species [3], but it also has been extended to other organisms such as plants [4,5]. Given a pan-genome of a species, then various subsets of genes in the pan-genome are of interest, such as the core genes, which are those genes that are present in all strains, the set of orphan genes that are present in only one strain (also called strain-specific genes), and the set of dispensable genes, which refers to genes that exist in a subset of the strains but neither in all nor just in one. So far, at least 17 microbial pan-genome projects have been conducted (see [6] for a review). Several scientific questions are followed using the pan-genome as a framework, such as the determination of genomic diversity of a species, reconstruction of the phylogenetic relationships between strains, or even to replace or at least question serotyping systems for the species in question [7]. The serotype is important for the epidemiologic classification of species and strains and it has great implications for decisions for example about medical treatment. Furthermore, pan-genomes play an increasing role in annotation efforts of bacteria. An example is the community wiki-type database *Aureo*Wiki at the University of Greifswald, which aims to unify gene/gene product information based on the pan-genome of *Staphylococcus aureus *(http://www.protecs.uni-greifswald.de/aureowiki). A unified nomenclature of genes, gene descriptions and gene names will help especially bacteriologists and life scientists to transfer knowledge from experiments with different strains of the same species on gene regulation, gene functions, mechanisms of pathogenicity and many more.

For the computation of a pan-genome most methods employ a BLAST-based approach or variants of it that compute orthologous gene groups. Orthologs are homologous genes that are related through speciation from a common ancestral gene, while paralogs have evolved through gene duplication. Widely used tools following this type of pan-genome implementation are PGAP [8], PanOCT [9], or PANGP [10]. BLAST-based approaches that do not take gene neighbourhood into account bear the risk of false orthologs clustering in particular of genes with many paralogs [11,9].

On the other hand in alignment-based approaches when genomes are compared based on genomic positions, typically a specific reference genome is assigned which acts as the coordinate system for the comparison. However, rearrangements and insertions or deletions lead to substantial architectural variations between genomes and therefore genomic regions that cannot be aligned to the reference are lost. We have proposed the SuperGenome [12] as a solution, which establishes a general global coordinate system for multiply aligned genomes. This enables the consistent placement of genome annotations in the presence of insertions, deletions, and rearrangements. From the SuperGenome the pan-genome can be computed in a straight-forward way. First, the start and stop positions of the annotated genes of the individual genomes are transferred into the shared coordinate system of the SuperGenome. After this, groups of genes are generated, depending if their annotations overlap in the SuperGenome. Finally, pairwise similarities of the overlapping genes are computed, which are used for the final grouping of the pan genes.

Also alignment-based methods that compute a pan-genome are not error-free. In particular regions with large sequence variation or with many copies of a gene class, such as tRNA gene clusters, the correct deduction of the pan genes is a challenge. For this, methods that visualise the gene order together with functional annotation can help to identify and possibly to resolve such cases.

Only few tools have been developed that explicitly address the task to visualise a pan-genome. A commonly used tool is the BLASTatlas [13], which maps and visualises whole genome homology of genes within a reference strain. Each genome in such a plot is represented as one circle with a unique colour, the intensity of the colour represents similarity with the respective orthologous gene in the pre-chosen reference genome. PGAT offers a web-based tool to support the homogenisation of genome annotation across the genomes of a species.

Many visualisations that are used for pan-genomes are not only static, but also mainly focus on visualising summary statistics. An example is the flower pot visualisation [14]. To our knowledge so far no tool includes analytical methods that can be triggered in connection with the visualisation, for example to (re)annotate genes of strains within a species. Here, we introduce Pan-Tetris, the first tool for interactive visualisation of pan-genome computation results. The pan-genome table is represented in a matrix-like visualisation with the aim to identify patterns of ordered pan gene groups which could be merged. Pan-Tetris offers such pan gene modifications by a Tetris-inspired interaction possibility.

We have applied Pan-Tetris to the visualisation of the pan-genome of the bacterium *Staphylococcus aureus*, a model organism for bacteriologists and life scientists.

## Methods

This section presents the specifications, design choices and realisation of Pan-Tetris, a framework for an interactive pan-genome map visualisation. A key aspect is the aggregation interaction technique that is implemented in Pan-Tetris to support user correction of the computed pan-genome. For this, we made use of the aggregation technique we have introduced in iHAT[15], here however, to support the interactive process of annotation-based pan-genome refinement.

### The SuperGenome-based pan-genome computation

Starting point of our visualisation is an alternative approach to computing a pan-genome. In contrast to reciprocal BLAST, we first compute a whole genome alignment (using progressiveMauve [16]) of the individual genome, from which we then build a SuperGenome. The SuperGenome provides a common coordinate system that allows a bidirectional mapping between the alignment coordinates and the original coordinates of each individual genome in the multiple genome alignment [12]. Next we compute the pan-genome based on the SuperGenome. For the computation of the pan genes we first note that in a multiple genome alignment orthologous genes if not too dissimilar will be commonly aligned, and secondly these will overlap in the coordinate system of the SuperGenome. The advantage of this alignment-based approach is that overlapping genes are more likely to be orthologs than paralogs, because of the synteny of the genes, that is implicitly taken into account while the multiple genome alignment is constructed. In addition, if the genes in the individual genomes have been annotated, the annotations can be directly transferred because of the bidirectional mapping provided by the SuperGenome.

Our method, which we coined 'PanGee' (unpublished software), computes the pan-genome from a SuperGenome of a multiple genome alignment. We define the pan-genome as computed by PanGee as the union of all genes that are contained in any of the individual genomes in the data set. It considers homologous relationships among these genes, which are represented by the computation of orthologous gene groups from genes that overlap in the coordinate system of the SuperGenome. In the context of PanGee and the underlying SuperGenome, a group of orthologous genes will be called a *pan gene*. A pan gene is defined as follows:

• it has a unique identifier;

• it contains at least one gene;

• it contains at most *n *genes;

• it cannot contain two or more genes from the same genome.

PanGee then outputs a pan-genome map which, similarly to other programs, reports the orthologous gene groups, i.e., all pan genes.

Another advantage is that due to the common coordinate system a specific ordering of the orthologs can be assigned based on the starting position in the alignment. This ordering gives a logical structuring of the groups without the need of a reference genome.

Nevertheless, a multiple genome alignment is in most cases heuristically computed. The non-optimality of such alignments can lead to erroneously aligned regions which can affect the pan genes' count. These erroneous regions are computationally difficult to detect. However, because of the logical ordering of the pan genes in the SuperGenome coordinate system, certain patterns in the absence and presence of genes within consecutive orthologous gene groups of the constructed pan-genome by PanGee give indications of these misaligned regions. A visualisation of this pan-genome can therefore help to identify these patterns and correct the errors caused by the alignment.

With this in mind we have developed Pan-Tetris. It uses the aggregation concept of our previously published tools iHAT[15], which we developed for the visualisation and analysis of genome wide association (GWA) data, and inPHAP[17], an interactive visualisation tool for genotype and phased haplotype data. The number of orthologous groups and therefore pan genes depend on the homologous relationships between the genes and the resulting multiple genome alignment.

### Graphical representation of the pan-genome

**Figure 1:**
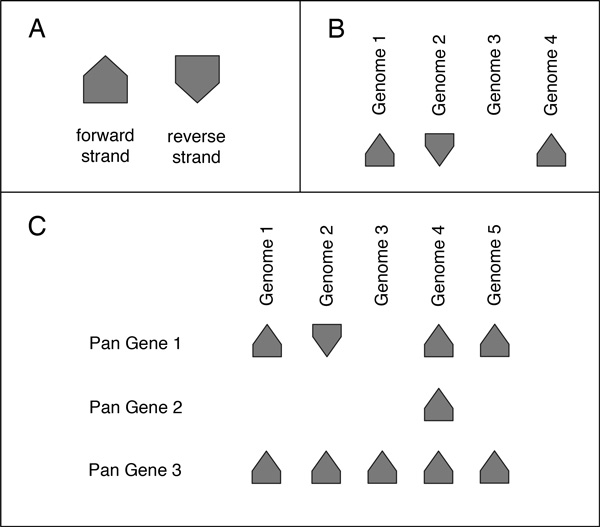
**Graphical components used in the visualisation of the **Pan-Tetris**pan-genome matrix**. A: Genes are represented by glyphs and build the basis of the matrix. Based on the strand information of the gene, a glyph can either point upwards (gene lies on the forward strand) or downwards (it is on the reverse strand). B: A pan gene, i.e., a group of orthologous genes is shown in a row. The presence and absence of genes, indicated by the presence and/or absence of the respective glyph in each column, reveals which individual genomes share or do not share an ortholog. C: The union of all pan genes constitute the pan-genome. This is represented as a matrix, which shows all pan genes in the rows, and the genomes in the columns. The pan genes are ascendingly ordered based on their appearance in the SuperGenome coordinate system.

Pan-Tetris features a matrix-like visualisation of the composition of all the pan genes of the pan-genome. Inspired by other visualisation tools like ConSet [18] it is basically a presence-absence visualisation of which individual genomes have a gene assigned to a pan gene. Thus, the matrix contains the entirety of genes (as cells) of all investigated strains (see Figure 1). The strains where the genes can be found are given as column titles. Gene occurrence is illustrated as clearly as possible by using equally sized and directed arrow shaped glyphs or if absent by using blanks. Glyph directions reflect the gene localisation on the forward or reverse strand of the DNA double helix. Rows summarize the genes of an orthologous group contributing to the definition of a pan-genome gene. Pan genes are given as row titles (first column of the matrix). By definition a pan gene can not contain two genes from the same genome. All pan genes in the computed pan-genome form the final level. Because of the SuperGenome coordinate system derived from the alignment every pan gene has a clearly defined start and stop position. Therefore, an ordering of the pan genes in their consecutive appearance in the SuperGenome is possible and as such visually presented to the user. The ordering of the pan genes is absolute as well as the ordering of the strains in the columns, which is defined by the ordering in the input file.

### Data formats and visualisation

**Figure 2:**
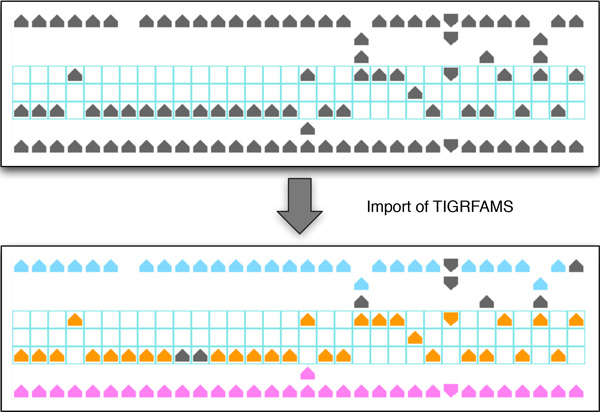
**Coloured encoding of functional gene annotation**. When a gene is present in a pan gene group it is represented by a unicoloured glyph. This glyph can be coloured based on TIGRFAM annotation, which can help to identify orthologous relationships, such as those that are highlighted. Though there are in principle hundreds to thousands of different TIGRFAM annotations, we restricted the colour encoding to 20 different colours chosen equidistantly from the HSB spectrum. Thus, a repetition of the colours may occur.

Pan-Tetris takes a so-called pan-genome map file, that is the typical output of PanGee. It is basically a tab-delimited file where each row refers to a pan gene, and most of the columns to strain-specific information (see Additional file S1 for an example). Furthermore, we made it possible to import data that has a very generic pan-genome format: this tab-separated data has to contain a header row with the corresponding genome names in each column, and rows with a unique identifier for each pan gene group. In addition to the primary pan-genome table data, we enable loading meta-information, such as further gene annotation descriptions. We have currently implemented the import of TIGRFAM assignments as meta-information. TIGRFAMs is a resource based on the use of HMMER3 [19] consisting of curated multiple sequence alignments, Hidden Markov Models (HMMs) for protein sequence classification, and associated information designed to support automated annotation of (mostly prokaryotic) proteins [20]. In alteration to the uni-colour display of a present gene in a pan gene group, genes can be coloured according to their TIGRFAM assignment (see Figure 2). Due to the large number of different TIGRFAM annotations, a unique colour mapping would lead to a wide colour spectrum, where the small nuance of the different colours could be hard to distinguish. Therefore, we restricted the colour encoding to 20 different colours that were chosen equidistantly from the HSB spectrum. To be aware that a repetition of the colours may occur, the TIGRFAM identifier is also added to the group description as additional information. If a gene has no TIGRFAM annotation, the default colour for a present gene is used. Besides the functional annotation colouring scheme, also a colouring of genes based on their location on either the forward or reverse strand has been implemented. This serves as an improved visual highlighting for the strand direction (an example is shown in the *Results *section). Over key-bindings the user can easily switch between the two gene colouring schemes.

The glyphs of each present gene within a pan gene for a specific genome are pre-rendered images to ensure a smooth interaction with the data. Also other graphical elements such as boxes to highlight selection of individual genes or rows and columns are pre-rendered. Due to this, all changes do not require a recalculation of the image, but instead just a repainting of the current view, which ensures a real-time response to user interaction.

### The Pan-Tetris graphical user interface

**Figure 3:**
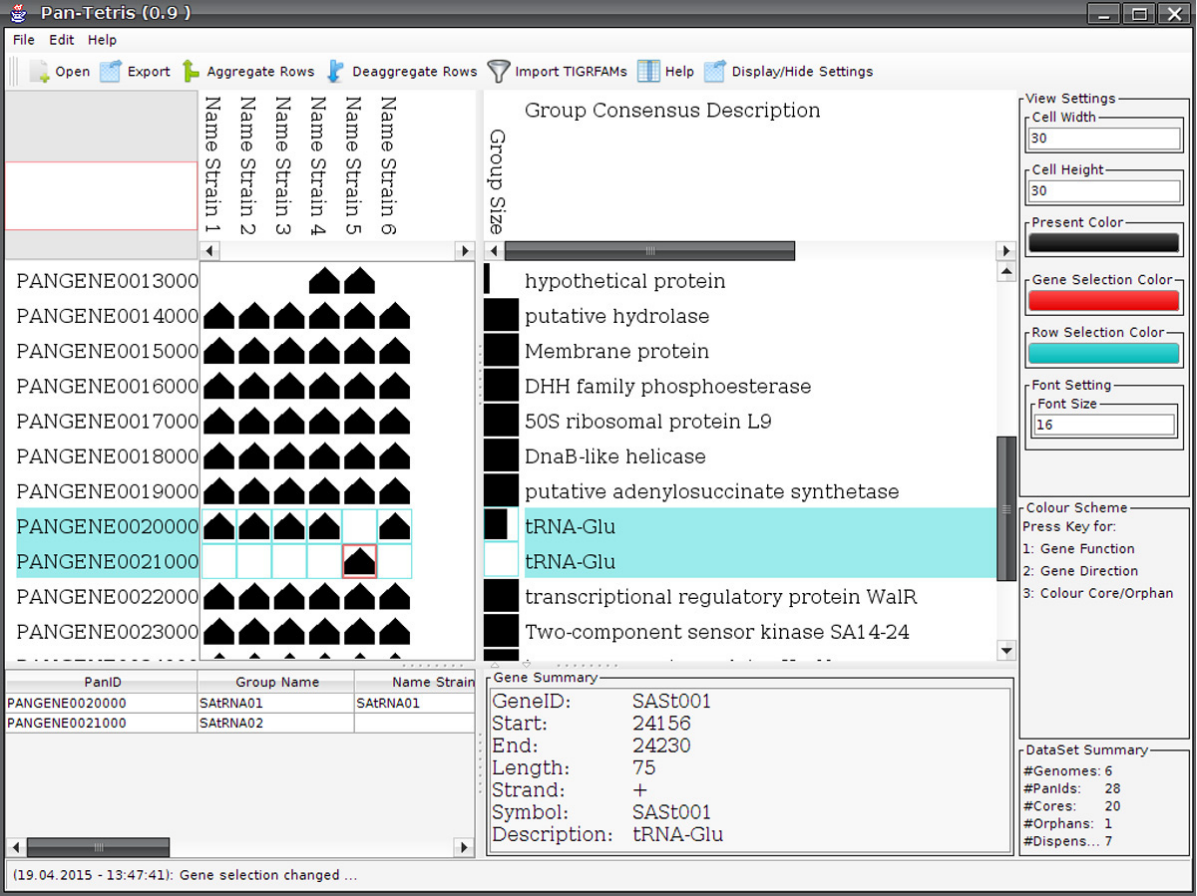
**Graphical user interface of **Pan-Tetris. The visualisation after a small pan-genome matrix (see additional file S1) has been loaded is shown. The upper left overview panel serves as an orientation map. The red outlined rectangle indicates which part of the complete matrix is loaded in the current view. Next to it the central pan-genome matrix visualisation is seen. Genes of a strain that are present in the respective pan gene group are shown by a glyph that either points upwards if the gene is on the positive strand and/or downwards if it resides on the negative strand. The column next to the pan-genome matrix visualisation is the colour encoding of the pan gene's group size, where core genes are coloured blue, orphans are coloured red and the size of the dispensable pan gene group is encoded by a gradient. The bottom panels are reserved for pan-gene annotations (left), gene annotation (middle) and summary statistics of the complete pan-genome loaded. Two rows have been selected in this example with the respective information shown in the left bottom panel, and one gene whose detailed information is shown in the middle bottom panel. The settings panel at the right allows the user to quickly change appearance of glyph sizes and colour encodings of the graphical elements.

The key feature of Pan-Tetris is to interactively assess and possibly change a pan-genome by visual means, i.e., to off an interactive possibility to correct pan gene computations. For this, we designed a graphical user interface (GUI) that consists of several components (see Figure 3). Intuitive user interface elements provide easy access to the individual functionalities of Pan-Tetris. The central and most important part of Pan-Tetris is the pan-genome matrix visualisation panel. It shows the absence and presence of genes in a matrix-like visualisation. In addition, pan gene identifier as row headers and the genome names as column headers provide further information. A detailed description of the visual representation of the pan-genome has been given in the *Graphical representation *section. The overview panel in the upper left serves as an interactive zoom out representation of the pan-genome visualisation. It shows the position of the current view in relation to the size of the pan-genome matrix. Next to the visualisation of the pan-genome, the description panel provides a consensus information for each pan gene, which is derived from the respective gene annotations. Furthermore, bar charts in this panel coloured black by default indicate number of genes that are contained in a pan gene. In addition, this black colour can be changed on demand to highlight core and orphan pan genes. A settings panel allows the user the customization of the visualisation. The bottom panels provide detailed information about selected rows in the pan-genome visualisation panel, gene meta-information of a selected gene and a general summary statistics of the loaded pan-genome. The latter includes the number of genomes, pan genes as well as core and orphan pan genes.

### Interaction possibilities

Pan-Tetris provides several possibilities of user interaction within the GUI which are described in detail in the next subsections.

#### General interactions with the GUI

In general, the number of pan genes in most pan-genome studies is very large in comparison to the number of genomes. For a fast navigation along these groups the user can also use the overview panel, which not only features the indication of the current view area by a red rectangle, but also to jump to a desired location (see Figure 3 for an example). Furthermore, it is possible to adjust the current view by changing the grid size, where the individual present genes are placed onto, or the colour of single graphical elements. The navigation through the graphical representation of the pan-genome is realized with navigation bars along the pan genes (vertical) as well as the genomes (horizontal).

#### Interactions with data

**Figure 4:**
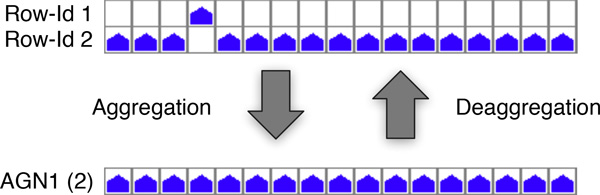
**Aggregation and deaggregation of pan genes**. The prerequisite for the aggregation is that the pan gene groups do not share genes from the same genome. The aggregation here is shown for two pan genes, which fulfil the prerequisite for being aggregated. Aggregation leads to fusing two or more pan genes into one common one. The aggregations can be interactively undone through the process of deaggregation.

A major feature of Pan-Tetris is to provide a clearly structured visualisation of the pan-genome matrix and to enable the user to assess possibly erroneously computed pan genes, that are the result of errors in the underlying multiple genome alignment with the possibility of a manual correction. Because of sequence diversity or other features, a pan gene can be disrupted into two or more pan gene groups. To facilitate the detection of such errors, protein sequence classifications (such as TIGRFAMs) can be used. Pan genes which are in direct or very close neighbourhood of each other in the SuperGenome, whose group compositions are complementary and that have the same functional annotation are an indication for disruption because of an erroneous alignment in that genomic region. In order to allow the user to interactively modify the composition of such pan gene groups, we implemented an aggregation technique, inspired by the famous Tetris game. Two or more pan gene groups that the user selected will be aggregated by merging these into a common pan gene group (see Figure 4). A pan gene group that results from aggregation gets an updated identifier labeled AGN, which is highlighted for easy visual detection, followed by the number of aggregated pan gene groups in brackets. In addition the identifier of aggregated groups is highlighted in the GUI. Of course, an aggregation is only possible when the pan gene groups to be fused are complementary, that is each genome has a gene in at most one of the pan gene groups. When this is not the case, the user will be informed that the aggregation is not feasible. The consistent location of the genes, whether on the forward or reverse strand, is not relevant for the aggregation of pan gene groups because orthologous genes can change their strand location due to an inversion event. After aggregation the number of pan genes and possibly the number of core and orphan genes will change. Therefore, the summary statistics is also updated. While the same data set is loaded, all aggregations of pan genes can be undone. Furthermore, Pan-Tetris includes an online documentation of last performed interactions and the possibility to save the complete history of all interactions.

By selection interaction the user can get further detailed information about the data. By selecting a pan gene (row), the meta-information of this group is displayed in the left bottom panel of the GUI. This information helps to provide a quick overview of the genes that are present in a specific pan gene group. Furthermore, it is possible to select single genes in the pan-genome visualisation panel. All available information about the respective gene is then displayed in the bottom middle panel.

To provide a convenient way of finding a gene, pan gene, aggregated groups or pan genes of specific function a search function has been implemented that allows the user to find, select and update the current view to the target location of those elements.

#### Export

Pan-Tetris provides two general export possibilities. Visualisations can be exported as publication-ready images either in bit-map formats (JPEG, PNG and TIFF) or as scalable vector graphics (SVG or PDF format). When the user modified the pan-genome matrix itself, this modified matrix can also be saved. The output format of the modified matrix is the same as the chosen input format.

### Supported platforms and availability

Pan-Tetris is written in Java 7, and can therefore be run on any machine with a Java VM installed. Pan-Tetris, including a tutorial video and example data, is available at http://bit.ly/1vVxYZT.

## Results and discussion

The development of Pan-Tetris is the result of a close collaboration with biologists who work on various pan-genome projects. No method for the computation of a pan-genome is error-free, and one of the aims for Pan-Tetris was to provide an interactive tool that offers the possibility to correct the computations of the pan-genome, which at the same time can then also be used to unify gene annotations. A correct pan-genome with a unified nomenclature of genes and gene descriptions is desirable and will help especially bacteriologists and life scientists to transfer knowledge on gene regulation and gene functions from experiments with different strains of the same species.

During our studies of the *Staphylococcus aureus *pan-genome we learned that lists and tables of orthologous genes alone are not suitable to describe the pan-genome of a bacterial species. The design choices were motivated by the inconvenient use of tables and their unsuitable depiction of possibly missed orthologous relationships. Due to this, we designed a simple visualisation that clearly separates individual genes and strains and at the same time allows the user to identify possible errors in the underlying pan-genome matrix.

The pan-genome as well as the pan gene concept is closely related to set-type data. Thus, our visualisation concept of Pan-Tetris is similar to set visualisation tools such as ConSet[18] and OnSet[21]. These tools let the user examine relationships between different sets with the help of basic set operations and aim at reducing large data sets with the focus to highlight differences and/or similarities between sets. While the aggregation approach of Pan-Tetris is in fact a specific type of a set operation, the focus of Pan-Tetris is, however the proof-reading of the output of an algorithm as well as curation of the data.

The resulting design of Pan-Tetris offers both an overview of the data as well as a possibility for a detailed inspection of the pan-genome matrix (see Figure 3).

The implementation of Pan-Tetris, in particular with its pre-rendered graphical elements provides a smooth navigation without noticeable loading times. Additionally, all interaction possibilities with data are intuitively and conveniently placed, which simplify the application for the user.

**Figure 5:**
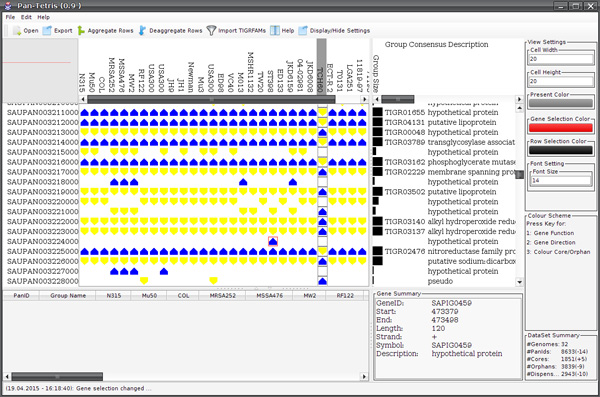
**The pan-genome map visualisation of *Staphylococcus aureus***. The map is shown with strand encoding, where in addition the direction colouring scheme was used. The strand encoding makes the inconsistent annotation of forward and reverse strand of the selected strain (TCH60) clearly visible. The size of the whole image of the pan-genome map can be estimated based on the current viewable area (indicated by red outlined rectangle) in the overview panel. After manual corrections (aggregations), the user is informed by the impact of the performed manual changes in the data summary panel in the lower right (number in brackets).

We have applied Pan-Tetris to the pan-genome of *Staphylococcus aureus*, a dangerous pathogen, that is a leading cause of bacterial infection in hospitals and in the community world-wide. At the same time *S. aureus *serves as a model organism to study the evolution of antibiotic resistance and pathogenicity. We have downloaded 32 whole genomes from GenBank (see Additional file S2) and computed the pan-genome of these genomes using our in-house developed software PanGee that uses our SuperGenome approach. The resulting pan-genome has 8647 pan genes, of which 1846 are core genes, 3848 are orphan genes, and 2953 genes belong to the dispensable genome. We then loaded the pan-genome map into Pan-Tetris (see Figure 5).

**Figure 6:**
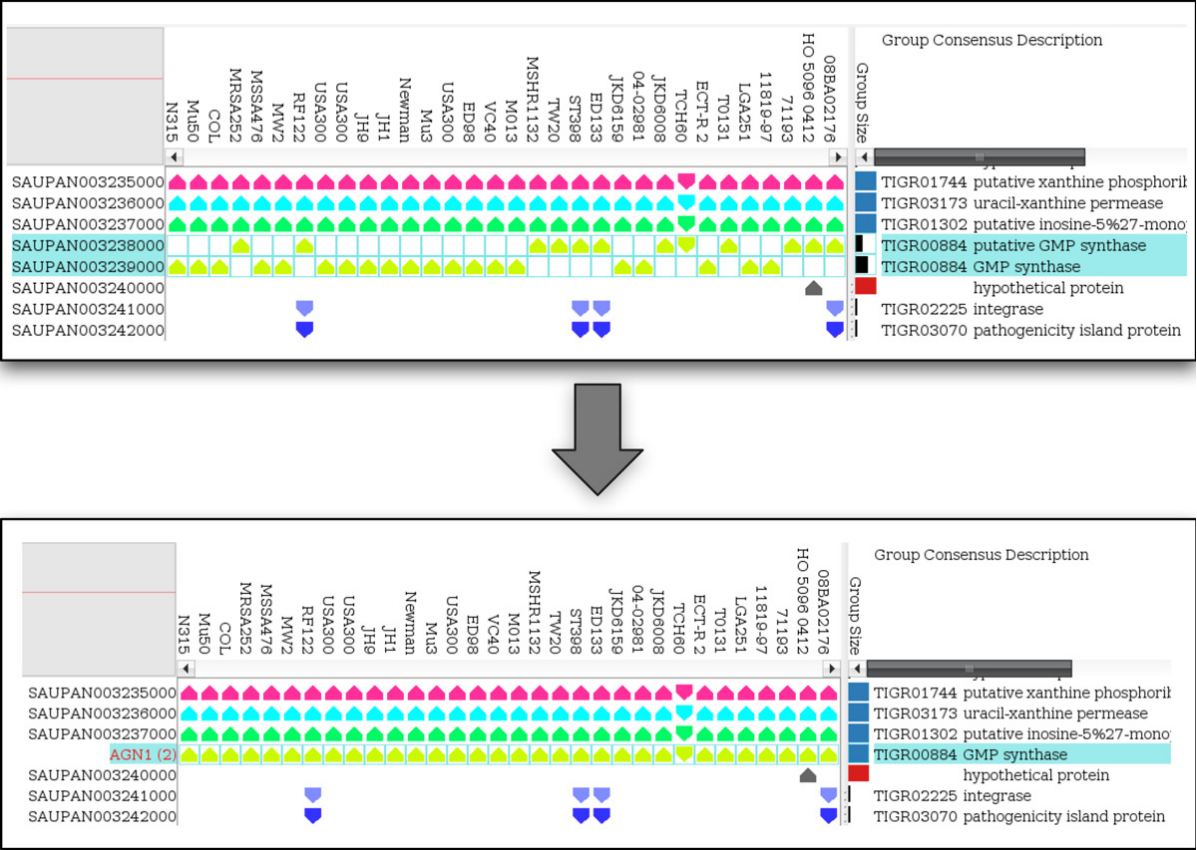
**The pan-genome map of *Staphylococcus aureus *used in conjunction with aggregation**. The upper part shows the pan-genome together with colouring of the genes based on TIGRFAM annotations, which are additionally displayed in the group description. Two rows have been selected, which are subject to aggregation, the result of which is shown in the lower part. In addition, the functionality of changing the group size scheme was used to highlight the orphan and core genes.

In addition to the pan-genome of *Staphylococcus aureus *we computed TIGRFAM annotations for all these genomes using HMMER3 [19]. These annotations were loaded in addition to the pan-genome and used in combination with the consensus gene description for the detection of erroneous grouped orthologs. The advantage of computing the pan-genome with the SuperGenome approach can be nicely demonstrated when viewing the matrix of the ordered pan genes together with functional assignment colouring. The import of TIGRFAM assignments colours the individual genes based on their respective meta-information. This visual support helps to detect possibly hidden orthologous relationships and therefore miscomputed pan gene compositions. With its Tetris-inspired aggregation technique Pan-Tetris supports the modification of these pan gene groups. An example for this is shown in Figure 6. Here, two pan gene groups with a common TIGRFAM colouring of the individual genes and the consensus description encode for a GMP synthase in both cases. Since also the gene neighbourhood is conserved (of which some are also core genes) as well as the gene direction, the two pan gene groups are nice candidates for aggregation. Indeed, after aggregation this group has become a core gene.

**Figure 7:**
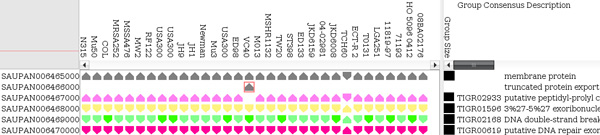
**Depiction of a truncated orphan gene from strain *VC40***. The next pan genes appears to be complementary to the orphan. Both groups are surrounded by core genes.

An additional typical use-case can be seen in Figure 7. Here, the apparently truncated gene *SAVC_08431 *in the strain *VC40 *appears as an orphan pan gene. In the following pan gene all other strains share orthologous genes. Since the consensus descriptions of both pan genes are basically identical and the two pan genes are surrounded by core genes, meaning that this genomic region is highly conserved, these two pan gene groups are good candidates for aggregation. It is not unusual that truncated genes are separated from their orthologs, because of their shorter sequence length, however, the occurrence of one truncated gene in an otherwise fully conserved region can be an indication for an erroneous annotation. After realigning the sequences of all genes of both pan gene groups it became obvious that *SAVC_08431 *was truncated because of an insertion of another adenine in a homopolymer segment. This lead to a reading frame shift and subsequently to a premature stop codon. It is known that sequencing errors often occur in homopolyers [22], thus a resequencing of the gene sequence is advisable. This example nicely demonstrates how the visualisation in Pan-Tetris can help to easily detect such possibly erroneous annotations.

Pan-Tetris is tightly linked to our method PanGee with which we compute a pan-genome of a data set. PanGee requires a multiple genome alignment, a possible drawback of our approach in comparison to the reciprocal BLAST based methods, since it is often a difficult endeavour to compute such a genome alignment. However, reciprocal BLAST approaches have no information about genomic rearrangements and are not robust against annotation errors, which makes a correction difficult.

Additionally, many labs sequence their own isolated strains and provide their own assembled and annotated genomes for databases. The problem is that independently performed genome annotations often result in variable gene start and end point predictions, varying gene lengths and often interfere with sequence errors resulting in the prediction in more or less truncated or multiple divided gene sequences. A reciprocal BLAST approach is here more likely to fail because the grouping of pan genes will be prevented because the found matches will not be sufficient to establish an orthologous relationship. In contrast to this the multiple genome aligner will still align truncated genes or place divergent gene sequences in direct neighbourhood to each other, which might lead to incomplete pan genes. An example for the notoriously difficult to align clusters of tRNA genes is shown in the supplement (see Additional file S3). With Pan-Tetris we offer an interactive possibility for correction. In total we identified many such or similar cases and using Pan-Tetris we were able to reduce the pan-genome of *Staphylococcus aureus *significantly. To this day, we know of no other tool that offers a refinement of a pan-genome.

### Future work

In the current version of Pan-Tetris we have concentrated primarily on a clearly structured visualisation of the pan-genome matrix computed from a multiple genome alignment using our SuperGenome approach to help improve a pan-genome and respective gene annotations. In a next version we will add further functionality, that for example allows the user to output just the core genome, the dispensable genome or only the orphans of the pan-genome of interest. For the research of mechanisms of pathogenicity, the core genome of an organism may reveal generic targets which can be suitable for a species but non-strain specific treatment (e.g., vaccines, antibiotics, cellular antagonist). Dispensable genes available only in subgroups of strains such as genes of mobile genetic elements, pathogenicity islands, plasmids or single genes of unknown origin may serve as strain specific markers for diagnostic purposes. They can be used to differentiate among phylogenetically related strain groups. Some of them are responsible for strain specific capabilities such as the resistance to antibiotics, synthesis of defined toxins, defined metabolic properties and further factors. The same is exclusively true for orphan genes but in a specific manner only for one of the analysed strains.

The next logical step that will improve the curation of pan-genomes is to connect Pan-Tetris with the underlying multiple genome alignment. Here, we plan to integrate a local multiple alignment method such as Clustal Omega [23] to realign candidates for aggregation.

There are a number of databases that offer precomputed pan-genomes of bacteria, a very prominent example is EDGAR[24]. Here, together with the developers and providers of EDGAR we plan to extend output formats such that users can visualize pan-genomes of EDGAR using Pan-Tetris.

Though traditionally defined for bacteria, the concept of the pan-genome can be and has been extended to other organisms, such as plants, where gene repertoire changes are observed. Pan-Tetris is not restricted to microbial species, however, as of right now it has only been tested for pan-genomes computed from multiple alignments of bacterial species. Last but not least, it is conceivable that pan-genome studies for closely related taxa could be performed at the nucleotide sequence rather than the gene level. Thus, using we could extend out SuperGenome approach and the computation of the pan-genome to general all orthologous sequence elements, revealing not only all protein coding sequences, but also non-protein coding features including promoters and small RNAs.

With these additional analytical functionalities we hope to make Pan-Tetris a truly powerful visual analytics tool for pan-genome computation.

## Conclusions

We have presented Pan-Tetris, a framework for the visualisation and interactive exploration of large-scale pan-genome matrices. With its close connection to our previously developed SuperGenome concept, a visual assessment of pan genes and the correction by aggregating different pan genes with common functional annotation is very straight-forward. To our knowledge Pan-Tetris so far is the only available interactive visualisation tool to explore and modify computed pan-genomes.

## Competing interests

The authors declare that they have no competing interests.

## Authors' contributions

JB created a first non-interactive visualisation for pan-genome data. AH and KN extended this idea and developed the concept of Pan-Tetris. The close collaboration of all authors lead to the designed graphical user interface of Pan-Tetris and the different visual encoding strategies. AH implemented Pan-Tetris in the Java(TM) programming language. AH and KN generated all used pan-genome data and investigated the orthologous gene groups. All authors wrote, read and approved the final manuscript.

## Supplementary Material

Additional File 1 Click here for file

Additional File 2 Click here for file

Additional File 3 Click here for file
