# Supplementary figures and images for: Pan-Tetris: an interactive visualisation for Pan-genomes
Source: BMC Bioinformatics. 2015 Aug 13;16(Suppl 11):S3. doi: 10.1186/1471-2105-16-S11-S3 (PMC4547177; doi:10.1186/1471-2105-16-S11-S3)

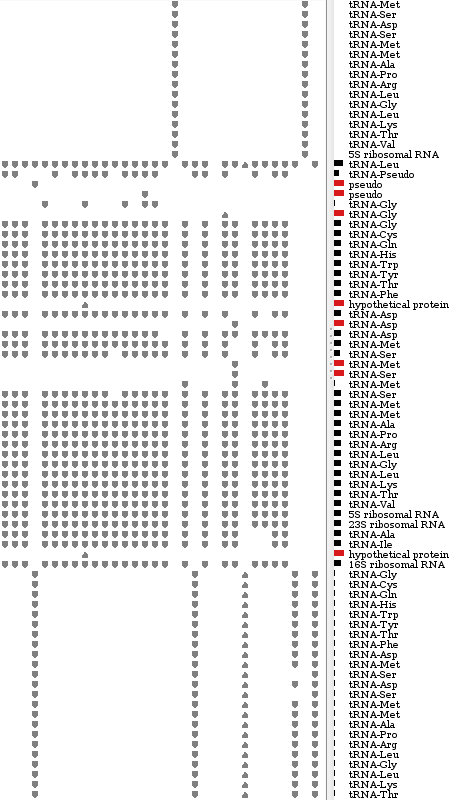

Supplement: Additional File 3 [file 1471-2105-16-S11-S3-S3.png]
